# The Application of Model Life Table Systems in China: Assessment of System Bias and Error

**DOI:** 10.3390/ijerph111212514

**Published:** 2014-12-01

**Authors:** Songbo Hu, Chuanhua Yu

**Affiliations:** 1 School of Public Health, Wuhan University, No. 115 Donghu Road, Wuhan 430071, China; E-Mail: husbo0910@sohu.com; 2 Global Health Institute, Wuhan University, No. 115 Donghu Road, Wuhan 430071, China

**Keywords:** model life table system, the infant mortality rate, adult mortality rate, the old mortality rate, life expectancy at birth, the average relative error

## Abstract

Model life table systems are extensively used in China in population mortality estimation and projection. Although China is the world's most populous country with approximately a fifth of the world's population, none of the empirical tables from mainland China were used in calibrating the existing models. In this paper, we applied recent three model life table systems with different inputs to China mortality data to investigate whether or not these systems truly reflect Chinese mortality epidemiological patterns and whether or not system biases exist. The resulting residuals show that, in most cases, the male infant mortality rate (1q0), adult mortality rate (45q15) and old age mortality rate (20q60) have a strong bias towards being overestimated and the life expectancy at birth (e0) bias is underestimated. We also give the detailed results for each case. Furthermore, we found that the average relative errors (AREs) for females are more than those for males for e0, 45q15 and 20q60, but for 1q0, males have larger AREs in the Wilmoth and Murray systems. We also found that the urban population has more errors than the rural population in almost all cases. Finally, by comparing the AREs with 10 other countries, we found the errors for China are more than those for other countries in most cases. It is concluded that these existing model life table systems cannot accurately reflect Chinese mortality epidemiological situations and trajectories. Therefore, model life tables should be used with caution when applied to China on the basis of 5q0.

## 1. Introduction

Life expectancy at birth (e0) and other summary indicators of mortality or longevity derived from life tables are key indicators of the health and well-being of a population. Life table calculations require a set of age-specific death rates, which are defined as the ratio of the numbers of deaths at each age in a given period, divided by the number of the average population-exposure for this age in the same period. The construction of a life table requires the assembly of available empirical demographic data, which are well known for their inaccuracies in less developed countries or areas because of the lack of high quality vital data registration systems. As a result, the construction of empirical life tables is not a straightforward procedure and model life table systems, which describe typical age patterns of human mortality, are used to estimate death rates indirectly for these countries or areas lacking reliable data.

Model life table systems are not constructed solely for the study of age patterns and mortality processes. They are used extensively in demographic and epidemiological analyses. Model life tables exploit the strong positive correlation between mortality levels at different ages as a means of predicting mortality levels for all ages using the limited information available. It is likely that one of the most important applications is to extrapolate complete age patterns, about which comparatively little is known in less developed countries, just from levels of child mortality such as 5q0, which are much more reliably documented [1], or from levels of child mortality and some other measure such as adult mortality 45q15. We begin with a brief review of existing model life table systems.

### 1.1. Overview of Model Life Tables

All model life table systems, which are not models in the usual sense of the word, are generated from the analysis of a large collection of historical mortality profiles. The central thesis of model life tables is that the complex phenomenon of age specific mortality rates can be adequately represented by one or several parameters, such as the family (or the pattern) to which the model belongs and the mortality level. All the classic and widely used model life table systems have two input parameters at most, which makes them simple and easily used in applied work.

There are eight major types of empirical models which are well-known and widely used: (I) Valaoras (1955); (II) Lederman (1969); (III) Coale and Demeny (1966/1983); (IV) Brass relational model (1971); (V) United Nations MLT for developing countries (1982; currently used by the United Nations Population Division (UNPD)); (VI) Murray *et al*. (2003; currently used by the WHO); (VII) Wilmoth *et al*. (2012), and (VIII) Clark and Sharrow (2012).

Model (I) is the first set of model life tables published by the United Nations in 1955 [2], which was computed by using quadratic regressions between q_x_ (probability of death) values of adjacent age groups starting from a specified value of infant mortality rate based on a collection of 158 tables for each sex. This set, subsequently published in a revised form [3], was a relatively simple one-parameter system indexed on infant mortality levels and produced a large cumulative error.

Model (II) was first published in 1959 and was subsequently revised over the course of the following decade [4]. This system is relatively complex, it contains univariate and bivariate models and provides some flexibility through a wider variety of entry values. However, most of these values are not easily estimated for most less-developed countries and the method is not easily used in practice.

Models (III) and (V) were the most widely used model life table systems in the past three decades. Both sets are the regional model life tables, which summarize variations in age-specific mortality using two dimensions: the “family” or the “pattern” which represents life tables with similar age patterns of mortality and the “level” which represents how age-specific mortality rates vary within a family as the overall level of mortality changes. Model (III) was first published in 1966 [5] and was subsequently revised in 1983 [6], based on a collection of 326 empirical life tables for both sexes, mostly from European developed countries. This model contains four families which were called: North, South, East, and West, and each family has 25 levels in the second edition. Moreover, this system is integrated in mortality estimation packages such as MortPak [7]. The system was updated in 1989, primarily to include extensions of the model life tables to age 100+ [8].

Model (V) is a revised set of United Nations model life tables for developing countries from 1982 [9], which is currently used by United Nations Population Division (UNPD). This system was computed by using a classical principal components analysis method based on a collection of 36 empirical life tables for each sex from less developed countries. Five family patterns—Latin American, Chilean, South Asian, Far Eastern and General—were identified in this model, each with a set of tables with a life expectancy ranging from 35 to 75 years for each sex.

The Brass system (model (IV)) used a different approach to constructing life table systems [10]. It is based on the assumption that two distinct age patterns of mortality can be related to each other by a linear relationship between the logits of their respective survivorship probabilities. Any life table can be related to a standard life table and two parameters through this transformation. The Murray system (model (VI)) is a modification of the two-parameter Brass relational model based on the shortcomings of the original Brass system, in that it has a systematic bias in its predictive ability as mortality levels depart from the standard [11]. The modified model incorporates two additional age-specific correction factors based on mortality levels among children and adults, relative to the standard. The modified logit system, which is currently used by the WHO, is a two-parameter system and is integrated in the Modmatch mortality estimation package [12].

The Wilmoth (model (VII)) and Clark (model (VIII)) systems are recent two-parameter model life table systems. The Wilmoth system was generated by using a log-quadratic model between the age-specific death rates (mx) and the probability of dying between birth and age 5 (5q0), and then by using a singular value decomposition method for the regression residuals [13]. The Clark system is a traditional model life table system with two parameters: families and levels [14], where five typical age patterns of mortality were identified by using the Model-based Clustering statistical technique. We need an single indicator of child mortality or the combination of a child indicator and adult mortality indicator to identify which family it should belongs to when applying this system to actual work through the related software packages [15]. Both these systems are based on a data collection from the Human Mortality database.

### 1.2. Problems with Applying Existing Systems to China

Model life table systems are used extensively in Chinese population mortality estimation and projection, despite known problems. Firstly, mortality registration and vital statistics system are incomplete and lack of uniform criteria in China. There are no reliable mortality data for some local regions, especially for the more economically backward areas, where under-reporting and misreporting of the birth and death dataset is more serious. Therefore, mortality estimation methods are urgently needed in these areas and model life table systems provide a good tool for studying demographic problems. Secondly, many early mortality data in China are lacking and not found. Sometimes, we need to estimate the age-specific mortality rates at earlier times. Thirdly, model life table techniques can be used to predict the future mortality. They are even used to evaluate the quality of mortality data for China [16]. Therefore, the assessment of the model life table systems’ application in China is needed.

China, with a population of 1.34 billion in 2013, is the world's most populous country, which makes up approximately a fifth of the world population. However, none of the empirical tables from mainland China was used by the aforementioned classic model life table systems in calibrating their models to produce model life table systems. There are three tables for the Hong Kong area used in the UN model life table system for developing countries (for each sex). In addition, the Wilmoth and Clark model life table systems both used the empirical tables from HMD, which includes the Taiwan area. However, fragmentary mortality data from the population of Taiwan and Hong Kong are nowhere near enough to represent most of the People’s Republic of China. The empirical tables used in the other model life table systems primarily come from European and American countries.

**Figure 1:**
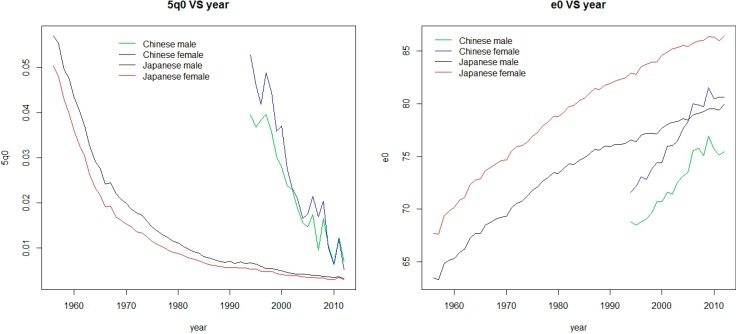
Comparisons of males and females between China and Japan for 5q0 and e0.

On the other hand, China has its own mortality characteristics that differ from those of most other countries. The first example is that the death rate had decreased rapidly during the past two decades. Table 1 presents the annual decrease rates of 5q0 and the annual increase ages of e0 for different countries for each sex (for the source of this collection see the next section). We highlight the maximum value of decrease rates of 5q0 and increase ages of e0. We can see that the Chinese annual decrease rates of 5q0 is 9.18% for males and 12.14% for females and the Chinese annual age increases of e0 are 0.367 for males and 0.502 for females, which are significantly higher than those of other countries. Another example is, in China, males have smaller 5q0 values than females, while the 5q0 values for males are larger than that for females in the vast majority of other countries in general. Figure 1 shows the 5q0 and e0 values during the period of 1994–2012 for China and during the period of 1956–2012 for Japan, which indicates two different points between China and Japan. Firstly, as in most countries, Japanese female 5q0s are always higher than the male values, while the converse is true for China. Secondly, the e0 increases with the decrease of 5q0 in general, as is shown for Japan between males and females. However, in China, the females have larger 5q0s (except for 2012) but still have the larger e0s.

Model life table systems are generated from a collection of empirical tables, which can only reflect the extent of mortality variation contained in the empirical data used to generate the system. There is a fundamental issue that we discuss in this paper when the classic model life table systems are applied to China: it is unclear that whether China’s mortality confirms to the patterns of existing classic model life table systems, or whether these existing systems reflect Chinese mortality epidemiological patterns, especially considering the different characteristics of mortality.

**Table 1:** The comparisons of annual decrease rates of 5q0 and annual increase ages of e0 between China and other countries by sex.

Country or Area	Period	n	5q0 Decrease Rates (%)			e0 Increase Ages	
			Male	Female		Male	Female
Bulgaria	1961–2010	50	2.72	2.89		0.037	0.103
Chile	1992–2005	14	4.48	4.51		0.268	0.249
Poland	1964–2009	46	4.58	4.10		0.126	0.183
China	**1994–2012**	**19**	**9.18**	**12.14**		**0.367**	**0.502**
Japan	1956–2012	57	5.05	4.95		0.294	0.335
Australia	1950–2009	60	3.03	3.03		0.224	0.210
Canada	1950–2009	60	3.71	3.49		0.218	0.217
Denmark	1950–2011	62	3.71	3.31		0.141	0.168
England and Wales	1950–2011	62	3.05	3.10		0.206	0.191
Italy	1964–2009	46	5.13	5.14		0.259	0.245
USA	1950–2010	61	2.72	2.32		0.183	0.169

Notes: (1) n is the length of the periods in the preceding column; (2) the decrease rates of 5q0 (r) for each country and sex are computed from the equation: q1(1 + r%)^n^ = q2, where q1 and q2 are the 5q0 values respectively in first and last year of the periods; (3) increase ages of e0 = (e02 − e01)/n, where e01 and e02 are the e0 values respectively in first and last year of the periods.

### 1.3. Purpose and Outline

Now that we know the possible problems that occur when the existing model life table systems are applied to China, we will investigate whether or not the biases exist in China and how the application in China performs relative to application in other countries. In this paper, we will apply three kinds of model life table systems to China and then assess the errors of these applications.

The rest of this paper is organized as follows: in Section 2, we provide an overview of the data used in this paper. In Section 3, we apply these model life table systems to China and describe how to implement the estimation programs. In Section 4, we present the methods used for assessing estimation errors. In Section 5, we give the results of the methods reported in Section 4. In Section 6, we discuss the results and provide our conclusions.

## 2. Data

The data used in this paper come from two main sources. First, we collected 19 (for each sex) period life tables for the total population from 1994 to 2012 from the China Population and Employment Statistics Yearbook of 1994–2012 edited by Chinese National Bureau of Statistics (this yearbook was called China Population Statistics Yearbook before 2007). In these yearbooks, the mortality for the total population was divided into three parts for each year and sex: city population, town population and rural population. We combine the city population and town population as urban population due to a lower mortality for some of ages. For each part of the population, we also collected 19 (for each sex) period life tables between the same periods.

All life tables were computed directly from observed deaths and average population counts of this yearbook without adjustment. All statistical data are divided into 20 age groups containing 0–1 age group, 1–4 age group and standard 5-year age categories with an open interval for age 90 and above, except for the years of 1995, 2000, 2005 and 2010, where the data are available up to an open interval of age 100 and above. The data from 1995 and 2005 is the data of a 1% nationwide sample and investigation. The data from 2000 and 2010 is from the Chinese fifth and sixth national census respectively. The rest is a 1‰ nationwide sample and investigation.

The second source is the Human Mortality Database (HMD), which is a publicly available dataset maintained by the University of California at Berkley (CA, USA) and Max Planck Institute for Demographic Research in Rostock, Germany [17]. We used period life tables covering 1-year time intervals from 10 different countries containing three less developed countries and seven developed countries from the HMD dataset. The earliest tables for many countries in HMD may date back to the 18th century and the most recent are from 2009–2012. The 5q0s in earlier times for these countries are far higher than that of 1994 for total population in China for each sex. Therefore, for each country, we included the starting year in which the observed 5q0 is similar to that of 1994 for the total population in China for each sex. This is because the application of model life table systems in this paper is on the basis of 5q0. The use of a similar range can make the comparisons more objective. However, it is impossible to choose the starting year in which the observed 5q0 is exactly the same as that for China, especially for both sexes. Therefore, the starting year chosen for each country is arbitrary at a certain range. Table 2 lists the range of 5q0 variation in these including periods for each sex and country, where the row above China is less developed countries and below is developed countries.

**Table 2:** The range of observed 5q0 for each country used in this study for each sex. Start means the value in the first year of the period and end refers to the value in last year of this period.

Country or Area	Period	Males			Females	
		Start	End		Start	End
Bulgaria	1961–2010	0.05062	0.01313		0.04134	0.00981
Chile	1992–2005	0.01941	0.01070		0.01602	0.00879
Poland	1964–2009	0.05706	0.00693		0.04557	0.00693
China	1994–2012	0.03946	0.00698		0.05280	0.00514
Japan	1956–2012	0.05708	0.00314		0.05036	0.00293
Australia	1950–2009	0.03476	0.00568		0.02715	0.00442
Canada	1950–2009	0.05472	0.00588		0.04314	0.00529
Denmark	1950–2011	0.04090	0.00408		0.03011	0.00388
England and Wales	1950–2011	0.03823	0.00578		0.03018	0.00442
Italy	1964–2009	0.04616	0.00431		0.03907	0.00364
USA	1950–2010	0.04130	0.00789		0.03230	0.00789

## 3. Applying the Model Life Table Systems to China

The recent three sets of model life table systems that we focus on in this paper are the Murray system (M), the Wilmoth system (W) and the Clark system (C). We do not focus on the Coale-Demeny and the UN model life table for developing countries, which are the commonly used model age patterns. This choice is motivated by three reasons: first, these three sets perform similarly and outperform the Coale-Demeny and UN model life table systems in estimation errors [13,14]. In addition, these two classic systems do not cover the entire range of epidemiological situations and trajectories between the probability of dying below age 1 and the probability of dying between age 1 and 5 [18]. Second, these three systems are able to reflect contemporary mortality experiences, including extremely low mortality levels. The Coale-Demeny and UN model life table systems which were constructed using the early datasets do not contain the lower mortality levels’ model life tables. Some of the mortality levels of the dataset we used lay outside the range of these two systems. For example, the life expectancy at birth of model life tables for the UN model life table for developing countries ranged from 35 to 75 years for each sex and pattern, but for the collection used in this paper, the range of the expectancy at birth is between 72 and 81 for Chinese females in the total population. Third, these three systems are simple and easily used for comparison, and they can be applied in practice for the same situations. For example, all the systems can be used based on the child mortality information such as 5q0 or 5q0 combined with 45q15.

We use these three systems to estimate complete life tables by sex for each year of the period 1994–2012 in China, respectively, from three cases of possible input parameters: case (1) 5q0 only, case (2) 5q0 & 45q15 and case (3) 5q0 & e0. The details in shorthand notation are shown in Table 3.

The application of these three sets needs child or infant mortality information. We focused on 5q0 rather than the infant mortality rate in the light of the fact that the infant mortality rate suffers from many more biases than 5q0 [18]. In many countries lacking vital registration (including China), under-five mortality is obtained from retrospective data, and the reporting of ages and dates often suffer of substantial transfers and heaping around age 1.

**Table 3:** The shorthand notation of model life table systems across three different input cases used in this study.

Model Life Table System	Case1: 5q0 only	Case2: 5q0&45q15	Case3:5q0&e0
Murray system (M)	M1	M2	M3
Wilmoth system (W)	W1	W2	W3
Clark system (C)	C1	C2 (level: 5q0); C2 (level: 45q15)	C3

In the Murray system, the calculation procedure can be implemented by Modmatch [12], which is a program for the Stata statistical software package. We can run this program by typing “modmatch” in the Stata command window, and then the user interface will appear. From this window, the complete model life table can be generated by inputting the values of 5q0, 5q0 & 45q15 or 5q0 & e0.

In the Wilmoth system, the mortality rate at age x (mx) can be related to two parameters (5q0 and *k*) through the following model, where the values of *a_x_*, *b_x_*, *c_x_* and *v_x_* can be estimated in two-stage procedure by fitting this model to HMD data [13]:

$$\text{log}(m_{x})=a_{x}+b_{x}\text{log}(5q0)+c_{x}{\left(\text{log}(5q0)\right)}^{2}+v_{x}k$$

From this model, a full life table can be derived given 5q0 and *k* values. In case1 only 5q0 is available, so *k* = 0 is assumed. In case2 (5q0 & 45q15) or case3 (5q0 & e0), an iterative procedure is required, in which we choose a value of k in order to reproduce the observed value of 45q15 or e0 exactly.

The Clark system is a little different from the other two systems which have two continuous parameters. The Clark system has a single continuous parameter (level) with five choices of ‘family’ variants (or pattern variants), like the Coale-Demeny and UN systems. In case C1 and C3, we use 5q0 to distinguish which family they should belong to, and then use 5q0 and e0 respectively as the “level” parameter. In case C2, the 5q0 combined with 45q15 is used to determine the appropriate family, but there are two options to determine the mortality levels: 5q0 and 45q15. We considered both of them and abbreviated them “C2(level: 5q0)” and “C2(level: 45q15)” respectively, as shown in Table 3. The calculation procedure can be implemented by a “LifeTables” package of R [15], which implements the HMD-calibrated version of their model for the R statistical package. By using this package, a full life table can be derived given either one or two pieces of information.

According to the programs described above, for each system and each case, 19 complete life tables by sex for the total population in China are estimated with the data described above. Similarly, complete life tables for the urban population and for the rural population are estimated. We now evaluate the errors for these estimation life tables and access the performance of these three model life table systems in China.

## 4. Methods of the Evaluation

In order to evaluate the estimation errors for full life tabled, four key mortality indexes are accepted: the infant mortality rate (1q0), the adult mortality rate (45q15), the old age mortality rate (20q60) and the life expectancy at birth (e0).

The estimation errors are assessed mainly from three aspects. Firstly, by analyzing the resulting residuals between observed values and estimated values for these four mortality indicators for each sex. Secondly, a further comparison for estimation errors is made between males and females and between the urban and rural populations, respectively. Thirdly, we compare the estimation errors to that of the other 10 countries presented in Table 2. The measure all the comparisons we use the average relative error (ARE) rather than the root-mean-squared errors considering the different mortality levels for each case.

## 5. Results

### 5.1. The Residuals of Applying Model Life Table Systems to China

**Figure 2:**
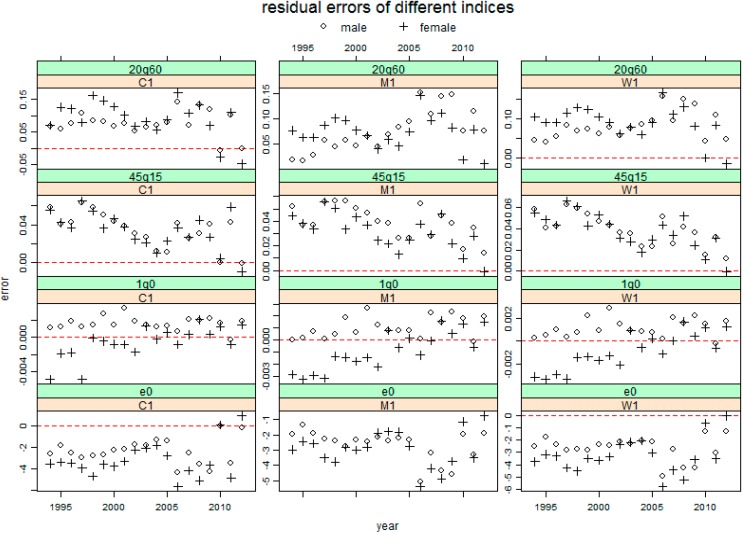
The resulting residuals for four mortality indicators predicting Chinese mortality by sex using three model life table systems respectively, given 5q0 only. The shorthand notations (C1, M1 and W1) were seen in Table 3. The red horizontal line represents 0 residual error.

**Figure 3:**
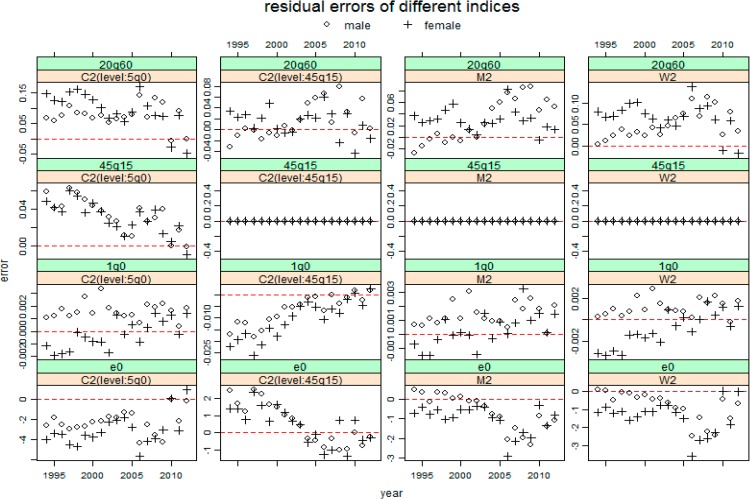
The resulting residuals for four mortality indicators predicting Chinese mortality by sex using three model life table systems respectively, given 5q0 and 45q15. The shorthand notations (C2((level: 5q0)), C2((level: 45q15)), M2 and W2) were seen in Table 3. The red horizontal line represents 0 residual error.

**Figure 4:**
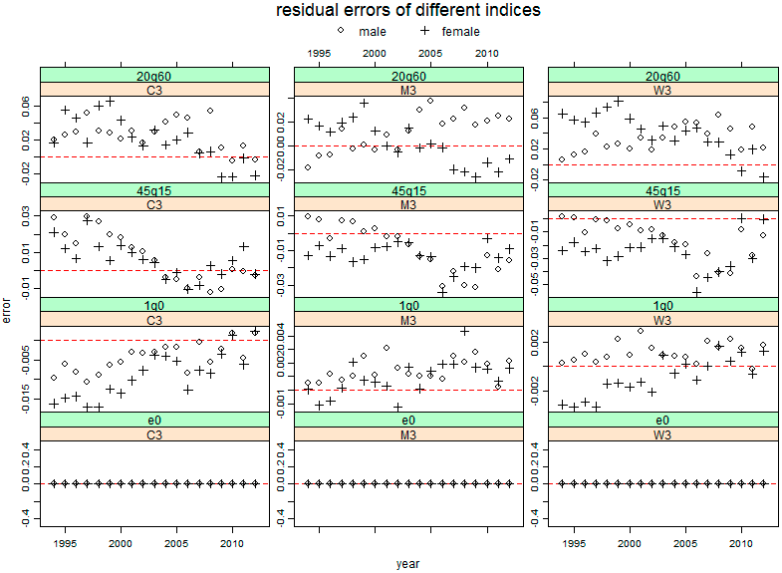
The resulting residuals for four mortality indicators predicting Chinese mortality by sex using three model life table systems respectively, given 5q0 and e0. The shorthand notations (C3, M3 and W3) were seen in Table 3. The red horizontal line represents 0 residual error.

Figure 2, Figure 3 and Figure 4 present the resulting residuals of the three cases with different inputs for the four mortality indicators e0, 1q0, 45q15 and 20q60 for total population by sex, respectively. In these figures, the residual for each year is represented by the circle for males and by the plus sign for females. These figures show that almost all the residual profiles for each sex are either below or above the horizontal line of residual 0, which indicates that applying these three model life table systems to China has a significant system bias.

Figure 2 shows that the residuals for each indicator have a similar profile using these three systems with 5q0 only as an input. First, 45q15 and 20q60 are overestimated and e0 is underestimated for each sex. Secondly, 1q0 is overestimated for males, but not for females for whom the residual errors appear to show a linear trend where the residual errors increase with the years.

Figure 3 shows the residuals using these three systems with 5q0 and 45q15 as inputs. The case “C2(level:5q0)” has a result roughly the same as that of case “C1”, which is not a surprise because the only difference between these two cases is that the case C1 uses 5q0 to determine the mortality family from five choices, while the case C2 uses 5q0 together with 45q15 to determine the mortality family. The other three cases are based on the level 45q15, which results in a residual value of 0 for each year. The M2 and W2 have similar residual profiles to case 1 for mortality indicators e0, 1q0 and 20q60, but with a smaller value than case 1 does. The “C2(level: 45q15)” is different from the M2 or W2 for indicators e0 and 1q0. However, most the residuals for 20q60 in case “C2(level: 45q15)” are overestimated as that in all other cases including the following case 3 in Figure 4.

Figure 4 shows the residuals using 5q0 and e0 as inputs. The 20q60s in this figure have the same results as above. Most residuals for 45q15 in M3 and W3 are negative, but in C3, the result is reversed. In addition, the 1q0 profile in M3 and W3 is different from that in C3 which is similar to that in “C2(level:45q15)”.

Table 4 summarizes these conclusions whether the results have a system bias for these four indicators predicting Chinese mortality using these model life table systems with different inputs, respectively.

Firstly, we see that the 20q60 has a strong bias towards overestimation. Secondly, except for C2(level: 45q15), the e0 bias underestimates for each sex. Thirdly, except for C2(level: 45q15) and C3, the 1q0 bias overestimates for males. Fourthly, the 45q15 bias overestimates except for case W3 and case M3, both of which bias underestimate. Furthermore, we find the “C2(level: 45q15)” and “C3” in Clark system are always different from other two systems with the same inputs’ situation, respectively, for e0, 1q0 and 45q15. This is because the Clark system is different from the Wilmoth and Murray systems as described in Section 3. The two continuous input parameters for the Wilmoth and Murray systems represent the mortality levels the estimating life table should have. However, for the Clark system there is only a single continuous level parameter. In “C2(level: 45q15)” and “C3”, the observed value is not equal to the estimated value for 5q0 which is used to choose the family and does not represent the mortality levels that the estimating life table should have.

We also analyze the resulting residuals respectively for urban population and for rural population, but we do not present these results in this study because both results are almost the same as those for the total population.

**Table 4:** A negative or positive system bias of the resulting residuals for four mortality indicators: e0, 1q0, 45q15 and 20q60.

Method	e0			1q0			45q15			20q60	
	Male	Female		Male	Female		Male	Female		Male	Female
C1	−	−		+	− +		+	+		+	+
W1	−	−		+	− +		+	+		+	+
M1	−	−		+	− +		+	+		+	+
C2(level:5q0)	−	−		+	− +		+	+		+	+
C2(level:45q15)	+ −	+ −		−	−		0	0		+	+
W2	−	−		+	− +		0	0		+	+
M2	−	−		+	− +		0	0		+	+
C3	0	0		−	−		+	+		+	+
W3	0	0		+	− +		−	−		+	+
M3	0	0		+	− +		−	−		+	+

Notes: (1) The “+” sign represents overestimate and the “−” represents underestimate. The “− +” represents the residual errors vary gradually from negative to positive and the “+ −” is reverse. 0 represents no error and also means the estimated value equals to observed value; (2) The shorthand notations for these methods were seen in Table 3.

### 5.2. The Comparisons between Different Sex and Different Population Respectively

Table 5 presents the resulting AREs for males and females for total population, where we also highlight the larger ARE values between males and females for each case and indicator. Firstly, we find the AREs of e0, 45q15 and 20q60 for females are always larger than those for males. This result can be explained by the facts shown in Figure 1. All the applications are based on 5q0 for which females have larger values than males, but for e0 females still have larger values. Secondly, the AREs of 1q0 for females are smaller than those for males in each cases except in C2(level: 45q15) and C3. In addition, the AREs for 1q0 in C2(level: 45q15) and C3 are significantly larger than those in others. The main reason is explained as before. 1q0 is related closely to 5q0. The estimated value for 5q0 equals to the observed value in all the cases except in C2(level: 45q15) and C3.

**Table 5:** The comparisons of AREs of the resulting residuals between males and females for four mortality indicators: e0, 1q0, 45q15 and 20q60 (%).

Method	e0			1q0			45q15			20q60	
	Male	Female		Male	Female		Male	Female		Male	Female
C1	3.3	**4.3**		**15.1**	9.2		24.7	**42.7**		14.7	**24.8**
W1	3.6	**4.3**		**12.9**	9.4		27.8	**45.1**		15.8	**22.3**
M1	3.6	**3.8**		**13.0**	9.9		28.7	**37.8**		15.4	**18.7**
C2(level:5q0)	3.1	**4.2**		**15.1**	8.1		23.6	**36.9**		14.6	**25.2**
C2(level:45q15)	1.5	**1.5**		34.6	**46.6**		--	--		5.0	**6.1**
W2	1.1	**1.8**		**12.9**	9.4		--	--		10.1	**17.3**
M2	1.1	**1.5**		**14.9**	8.9		--	--		7.5	**7.6**
C3	--	--		29.3	**44.9**		8.5	**10.4**		5.0	**6.5**
W3	--	--		**12.9**	9.4		12.5	**32.6**		6.4	**10.2**
M3	--	--		**15.7**	11.5		10.1	**17.5**		3.2	**3.7**

Notes: (1) “--”represents 0 error and also means the estimated value equals to observed value; (2) The shorthand notations for these methods were seen in Table 3.

In Table 6, we compare the resulting AREs between urban population and rural population for each sex, where we also highlight the larger ARE values between urban population and rural population for each case and indicator. We find that the urban population data has larger errors than the rural population almost for all the cases and indicators.

**Table 6:** The comparisons of AREs of the resulting residuals between urban population and rural population for four mortality indicators: e0, 1q0, 45q15 and 20q60 (%).

Method	e0			1q0			45q15			20q60	
	Urban	Rural		Urban	Rural		Urban	Rural		Urban	Rural
**Males**											
C1	**3.6**	2.4		**18.3**	15.9		**33.9**	18.5		**15.1**	11.6
W1	**4.4**	2.5		**16.6**	14.2		**40.6**	18.3		**20.8**	10.8
M1	**5.0**	2.2		**19.0**	14.1		**42.9**	17.6		**24.8**	9.3
C2(level:5q0)	**3.3**	2.6		**19.9**	15.8		**29.2**	18.6		**14.8**	11.8
C2(level:45q15)	1.0	**1.6**		29.4	**33.5**		--	--		4.3	**5.2**
W2	**1.6**	0.8		**16.6**	14.2		--	--		**12.4**	7.8
M2	**1.5**	0.8		**20.9**	14.8		--	--		**10.1**	6.8
C3	--	--		**29.3**	22.0		**9.4**	8.2		3.7	**5.8**
W3	--	--		**16.6**	14.2		**21.0**	7.6		5.3	**6.0**
M3	--	--		**21.9**	15.4		**15.5**	7.3		3.4	**4.2**
**Female**											
C1	**3.7**	3.7		**13.8**	11.4		**44.8**	30.2		**22.9**	18.6
W1	**4.0**	3.8		**11.9**	10.9		**52.1**	34.2		**24.2**	18.0
M1	**4.1**	3.0		**13.3**	10.9		**47.3**	27.4		**24.5**	13.4
C2(level:5q0)	3.6	**3.9**		**14.1**	9.1		**43.2**	28.4		21.5	**22.8**
C2(level:45q15)	0.9	**1.2**		**51.3**	42.2		--	--		**7.2**	6.7
W2	**2.1**	1.4		**11.9**	10.9		--	--		**17.8**	14.2
M2	**1.7**	1.0		**16.1**	9.1		--	--		**7.6**	6.5
C3	--	--		**49.9**	37.9		**9.8**	9.6		**7.5**	5.8
W3	--	--		**11.9**	10.9		**39.3**	22.0		8.2	**10.5**
M3	--	--		**22.1**	9.7		**25.8**	12.1		**8.0**	3.1

Notes: (1) “--“represents 0 error and also means the estimated value equals to observed value; (2) The shorthand notations for these methods were seen in Table 3.

### 5.3. The Comparison with Different Countries

Similar to the application in China, we also apply these three model life table systems to 10 other countries presented in Table 2 for each sex, and then compute the AREs of these indicators for each country and case. Table 7 lists the AREs of e0, where China refers to total population. For each case, we highlight each ARE value that is more than that for China. In addition, we highlight the row of China for each sex. We see that, for the case C1, W1, M1 and C2(level 5q0), the AREs in China are significantly more than those in any other countries for each sex. For other cases, there are only a few exceptions that the AREs in China are smaller. However, generally, the model life tables perform worse in China. The other three indicators (1q0, 45q15 and 20q60) also have similar results, all of which indicate that the applications of these model life table systems perform worse in China than in other countries.

**Table 7:** AREs of observed e0 for different countries using three model life table systems with different inputs, by sex (%)

Country or Area	C1	W1	M1	C2(level:5q0)	C2((level:45q15)	W2	M2
**Male**							
Bulgaria	3.1	2.7	2.5	3.1	**2.7**	0.3	0.5
Chile	1.9	2.0	2.3	2.4	0.8	**1.5**	**1.5**
Poland	3.3	3.2	2.9	2.2	1.5	0.4	0.6
**China**	**3.3**	**3.6**	**3.6**	**3.1**	**1.5**	**1.1**	**1.1**
Japan	1.1	1.8	2.6	1.1	0.9	1.0	**1.4**
Australia	1.8	2.0	2.3	1.8	0.7	0.7	0.8
Canada	1.6	1.9	2.0	1.6	0.7	0.8	0.8
Demark	1.8	1.7	1.6	1.8	0.9	0.5	0.4
England and Wales	0.9	1.1	1.4	0.9	1.4	1.0	1.1
Italy	1.4	1.9	2.3	1.4	0.8	0.4	0.5
USA	1.3	1.3	1.5	1.4	**2.5**	1.1	**1.2**
**Female**							
Bulgaria	1.4	1.2	1.2	1.4	1.5	1.1	1.2
Chile	2.8	2.4	2.4	2.3	1.0	1.7	**1.7**
Poland	1.2	1.0	0.7	1.1	0.8	0.3	0.4
**China**	**4.3**	**4.3**	**3.8**	**4.2**	**1.5**	**1.8**	**1.5**
Japan	1.5	2.0	2.8	1.5	1.2	1.5	**1.8**
Australia	1.1	1.3	1.7	1.0	1.0	0.8	1.0
Canada	1.6	2.0	2.0	1.6	1.2	1.4	1.5
Demark	1.6	1.1	1.0	1.4	0.8	0.5	0.9
England and Wales	0.6	0.5	0.8	0.6	0.6	0.4	0.6
Italy	1.9	2.3	2.5	1.8	0.8	0.9	0.8
U.S.A.	0.8	0.8	0.9	1.0	**2.4**	1.4	**1.8**

Notes: The shorthand notations (C1,W1,…) were seen in Table 3.

## 6. Discussions and Conclusions

In this paper, three recent model life table systems are applied to China, for the total population, the urban population and the rural population, respectively. Each system is used to estimate a complete life table on the basis of three cases of possible input parameters: 5q0 only, 5q0 combined with 45q15 and 5q0 combined with e0. By analyzing the resulting residual errors for the four key mortality indicators e0, 1q0, 45q15 and 20q60, we found these applications have a significant system bias in China. In addition, by comparing the AREs between males and females, we found the errors of e0, 45q15 and 20q60 for females are always more than those for males, but 1q0s for males have larger errors except for the Clark system (C2(level:45q15) and C3). Furthermore, we found the urban population has larger errors for each sex, compared with the rural population. Finally, we found the errors in China are more than those in other countries.

The main reason why the applications of model life table systems in China contain a significant system bias and perform worse than that in other countries is that in the construction of these three systems no mortality data from the mainland of China where the mortality patterns are different from most other countries were used.

**Figure 5:**
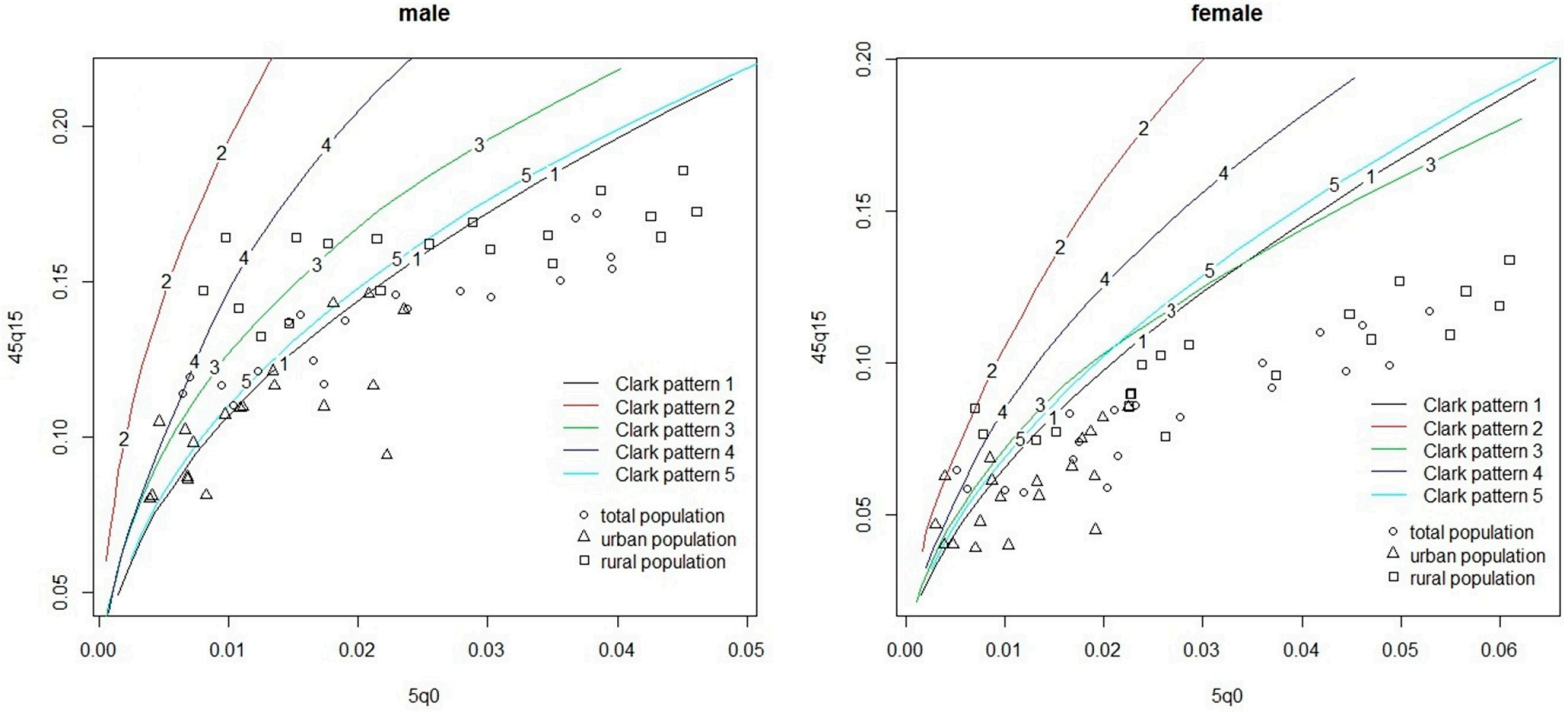
Relationship between 5q0 and 45q15 in population-years of the data from China. Five kind of different color’s lines represent the five different mortality patterns for the Clark model life table system.

We take the Clark model life table system and look at its relationship between 5q0 and 45q15 as an example of its worse performance in China. Figure 5 presents the 5q0 *versus* 45q15 relationship for each sex in China from the data used in this paper, together with the 5q0 versus 45q15 relationship modeled using the Clark model system. The percentages and estimation errors for observed values of 45q15 that fall outside the range estimated by Clark system on the basis of level 5q0 are summarized in Table 8, where we also present the results for Japan and the U.S.A.

**Table 8:** The percentages and estimation errors for observed values of 45q15 that fall outside the range estimated by the Clark system, with 5q0 as an input (%).

Country or Area	n	Percentage below Range	Percentage above Range	Percentage outside Range	ARE below Range	ARE above Range	ARE outside Range
**Male**							
Whole China	19	63.2	0.0	63.2	29.8	--	29.8
Urban China	19	63.2	0.0	63.2	43.7	--	43.7
Rural China	19	52.6	0.0	52.6	25.7	--	25.7
Japan	57	0.0	0.0	0.0	--	--	--
U.S.A	61	0.0	0.0	0.0	--	--	--
**Female**							
Whole China	19	89.5	0.0	89.5	46.4	--	46.4
Urban China	19	73.7	5.3	78.9	53.8	24.0	51.8
Rural China	19	89.5	0.0	89.5	32.4	--	32.4
Japan	57	0.0	0.0	0.0	--	--	--
U.S.A	61	0.0	0.0	0.0	--	--	--

Notes: (1) Below range means the observed 45q15 below the minimum model-estimated value. Above range means the observed 45q15 above the maximum model-estimated value. Outside range means either below range or above range; (2) Whole China represents the total population in China.

Figure 5 shows that most of the data points represented for China fit remarkably badly in comparison within the range modeled by the Clark model life table system, especially for females. From Table 8, comparing with Japan and U.S.A both of which have no data points outside the range modeled by Clark system, at least more than half of data points for China are outside the range for each case. All of these explain the poor performance in applying model life table systems to China. Moreover, except for 5.3% for urban China/females above the range, all the data points outside the range are below the range, which indicates the overestimate for 45q15 and the significant system biases.

There are almost no overall differences for the percentages outside the range between the urban and rural populations (the percentage for urban males is about 11% more than that for rural males, but it is the converse for females). However, the AREs of the urban population are larger than that of the rural population, mainly due to the lower mortality levels of the urban population.

This example not only explains the reason why the model life table systems perform so poorly in capturing Chinese age-specific mortality, but also reflect the different mortality patterns for China, which perhaps is due to China’s own mortality characteristics. China’s economy has developed rapidly with a rapid decline of the mortality rate over the last thirty years. In addition, China has a large population and wide range territory with many races, which result in uneven development. There are big differences in culture and social customs among different races and regions. The mortality conditions may also differ from region to region due to the differences in geographical environment and economic development.

In fact, China has his own model life table system, which was developed by Jiang [19,20]. This model life table system has a typical two-dimensional variation: the ‘‘family’’ and the ‘‘level’’. Like the UN model life table for developing countries, five families for this system were determined on the basis of the collection of data mainly from the 1982 China population census. This system was first published in 1990, where 36 model life tables with integral digit e0 from 40 to 75 years and life tables were developed for each sex. However, since this system shows similar deficiencies as the Coale-Demeny system and the UN model life table system for developing countries (explained in Section 3), this system was not included in this study.

In this paper, we show the system bias in applying recent model life table systems to China. The results of these biases for each indicator may help whoever wants to apply these systems to China. This also suggests that the existing model life tables do not cover the entire global range of epidemiological situations and trajectories. Therefore, model life tables should be used with caution in applying to China on the basis of 5q0.

All the application procedures of model life table systems in this paper are on the basis of 5q0 as input parameter at least. The evaluation of these applications assumes that the common observed value of 5q0 is correct, which may not always be warranted, especially for some backward regions. On the other hand, the application of model life table systems can be based on other input parameters, such as 1q0 or 1q0 together with other parameters, which we didn’t discuss in this paper. Therefore, a systematic evaluation for the application of model life table systems in China is urgently needed, along with the development of new model life table systems that could cover a wider range of epidemiological situations and trajectories including that of China.
